# Lethal and sublethal toxicity of ytterbium in rainbow trout juveniles

**DOI:** 10.1007/s11356-025-36315-5

**Published:** 2025-03-31

**Authors:** Joëlle Auclair, Eva Roubeau-Dumont, Chantale André, François Gagné

**Affiliations:** Aquatic Contaminants Research Division, Environnement and Climate Change Canada, 105 McGill, Montréal, Québec H2Y 2E7 Canada

**Keywords:** Ytterbium, DNA damage, Protein aggregation (amyloids), Thioneins, Lipid peroxidation

## Abstract

The heavy rare earth element ytterbium (Yb) is a critical element of technology finding its way into urban wastewaters from solid waste disposal sites. The purpose of this study was to investigate the lethal and sublethal toxicity of Yb to rainbow trout juveniles. They were exposed to increasing concentrations of Yb^3+^ (0.06–40 mg/L) for 96 h at 15 °C. Mortality was recorded, and in the surviving fish, the following biomarkers were determined: protein aggregation, thioneins, lipid peroxidation (LPO), and DNA damage in gills and the liver. The 96-h lethal concentration (LC50) was 2.7 ± 0.66 mg/L indicating that this element is toxic to fish. Biomarker responses to Yb occurred at concentration 45 times less than the LC50 in some cases. The following biomarkers were positively (*p* < 0.05) correlated to fish survival: liver thioneins, gill DNA strand breaks, LPO, and protein aggregation in the liver. The decrease in LPO in the liver and gills by Yb suggests reduced production of reactive oxygen species production. In conclusion, Yb is toxic to trout juveniles producing sublethal effects at concentrations range of 60 µg/L after 96 h. This concentration represents an upper limit for consideration for aquatic animal health.

## Introduction

Rare earth elements (REEs) are a family of 15 elements that display homogenous physicochemical properties (Cotton 2006). For example, they adopt primarily the 3^+^ oxidation state with similar ionic radii (Emsley 1989). The ionic radii decrease with the atomic number and tend to precipitate at neutral pH in the form of metal oxides. Lanthanides are considered critical elements of technology widely used in many important sectors of our economy including healthcare and energy production, optoelectronics (lasers), luminescent probes including emerging low-carbon technologies (Pallares et al. 2020; Chen et al. 2020). Gadolinium-based compounds are used as contrast agents for magnetic resonance imaging while others, like ytterbium (Yb), are being explored as radiosensitizers (Khaydukov et al. 2016). Yb is considered as another contrast agent for X-ray computed tomography imaging. Yb has K-edge energy (61 kev compared to 33 kev with iodine chelates) that is located just within the higher-intensity region of X-ray spectra, which can induce significant enhancement of image contrast for optimal image diagnostics (Liu et al. 2014). Notwithstanding this, no increases of Yb in the dissolved fraction was observed as with Gd-based contrast agent albeit the heavy REEs in the dissolved fraction tend to increase in wastewaters (Turcotte et al. 2022). Heavy REEs are also considered assets for waste conversion and resource recovery. For example, the recycling of waste cardboard was enhanced by erbium and Yb triflates (Yb(OTf)_3_) using hydrothermal decomposition (Kim et al. 2022). Therefore, there is a need to fully understand their potential effects in ecosystems to ensure the safe and sustainable use of these elements. Only few reports on the presence of Yb in potential sources of contamination such as solid wastes leachates, street runoffs, and wastewaters are found in the literature. Its total concentrations range between 1 and 10 ng/L in treated wastewaters, and its concentration could increase to 30 ng/L in untreated effluents (Turcotte et al. 2022). It is estimated that billion of liters (L) of effluent are released every day in Canada (Environment and Climate Change Canada, 2023) representing at least 1–10 g of Yb being released each day. Because effluents are continuously released in the aquatic environment and the increasing use and inappropriate disposal of the critical elements of technology in solid waste runoffs/leachates, the toxicity of Yb should be examined more closely for the protection of aquatic ecosystems.

The toxicity of Yb in aquatic animals has not been well explored at present. In the goldfish *Carassius auratus*, a strong inhibition in catalase activity was observed at 0.05 mg/L of Yb^3+^ after 40 days (Hongyan et al. 2002). Glutathione (GSH)-dependent enzymes such as GSH S-transferase and peroxidase were also decreased at 0.1 mg/L suggesting altered GSH metabolism by Yb. Yb was found to have very high binding affinity to reduced GSH, thereby depleting it from the enzymes above (Garg et al. 2003). Moreover, REEs with high GSH binding activity were shown to decrease metallothioneins levels in fish hepatocytes, thereby increasing susceptibility to heavy metals and oxidative stress (Hanana et al. 2021a). The effects of lutetium (Lu), one atomic number higher than Yb, also had no significant influence on metallothionein levels suggesting a similar mechanism (Hanana et a., 2021b). The trivalent nature of REE could also interfere with iron/heme metabolism, which have the trivalent iron element involved in oxygen binding (Ghio et al. 2011). For example, *Oncorhynchus mykiss* trout exposed to Lu had elevated gene expression of ferritin, a major iron-regulating transporter in cells (Hanana et a., 2021b). Both Yb and lanthanum (La) disrupted heme homeostasis and root architecture in *Arabidopsis thaliana* (Grosjean et al. 2024). Exposure of zebrafish embryos to Yb^3+^ delayed embryo and larval development (Cui et al. 2012). At concentration of 17 mg/L, Yb^3+^ and La^3+^ increased larval mortality to 80 and 30%, respectively, indicating that the heavier Yb is more toxic than the light REE La. In another study, the toxicity of 15 lanthanides were examined in fish cell lines revealing that many of the heavy REEs, including Yb, were generally more toxic (Fleurbaix et al. 2022). In freshwater bivalves exposed to 1 mg/L to each of Yb, Gd, and Nd for 96 h, significant accumulation in tissues was observed mainly in gills and digestive gland (Lachaux et al. 2023). The estimated bioconcentration factor (tissue levels of element/exposure concentration in water) for Yb ranged from 49 to 99 indicating that this element is bioavailable. Recent evidenced that heavy REE could also induce oxidative stress leading to lipid peroxidation and DNA damage (Hanana et al. 2024). The accumulation of lipid and DNA damage in tissues could lead to decreased survival, growth, and reproductive success in aquatic animals (Lacaze et al. 2011). The interaction of REE on proteins could also lead to denaturation and degradation contributing to the formation of age-related pigments (lipofuscin)s and amyloids (Cabaleiro-Lago et al. 2012). Amyloids are proteins with β-sheet conformation leading to insoluble aggregates forming plaques that accumulate in tissues leading to the gradual dysfunction (Alhazmi and Al-Bratty 2017). Hence, the need to examine the mode of action of Yb in fish to better understand the environmental risk of this critical element of technology.

The purpose of this study was therefore to investigate the sublethal and lethal toxicity of Yb in juvenile (*Oncorhynchus mykiss*) rainbow trout. The lethal concentration (LC50) was determined in trout yearlings, and the sublethal toxicity was examined in the surviving fish in gills and liver. Sublethal effects were determined with the following biomarkers at increasing levels of biological complexity from metal binding thioneins, protein aggregation (amyloids), oxidative stress (lipid peroxidation), DNA damage, gill/liver mass index, and fish condition. These effects are consistent with the reported impacts found in the literature for heavy REEs (Hanana et al., 2021a, b, c, Fleurbaix et al. 2022; Dubé et al. 2019; Constantin et al. 2024). The mode of action of Yb was discussed considering the observed sublethal effects, and an attempt was made to relate those with fish survival.

## Methods

### Sample preparation and exposure to rainbow trout juveniles

Yb trichloride (YbCl_3_) was purchased as powder from Sigma Aldrich (On, Canada) and dissolved in MilliQ water at 100 mg/L concentration. Yb is highly soluble in water with an estimated solubility of 170 g/L (Judge et al. 2023; Constantin et al. 2024). The conductivity (conductivity meter, Thermofisher Scientific) of the stock solution at 100 mg/L was measured following 1 and 96 h dissolution in MilliQ water and aquarium water revealed no loss of ion activity. *Oncorhynchus mykiss* trout juveniles were collected from a local hatchery (Sherbrooke, Québec) and acclimated to our laboratory for 1 month before initiating the exposure experiment following recommended guidelines from the animal care committee of Canada. The aquarium water was dechlorinated and UV-treated tap water (pH 7.8–8.2; conductivity: 240–280 uScm^−1^; organic carbon content < 0.8 mg/L) from the city of Montreal and was maintained in 300 L tanks at 15 °C under constant aeration. The fish were feed once daily with commercial trout chow. The young fish were exposed to increasing concentrations of Yb following a standard methodology (Environment Canada 2007). Briefly, 10 yearlings (0.4–2.3 g wet weight) were placed in 60 L of aquarium water (charcoal and UV-treated tap water) in plastic containers lined with polyethylene sacs with aeration. The fish were thus exposed to increasing concentrations of Yb using a semi-logarithmic (five-fold) increment starting at 0.06, 0.32, 1.6, 8, and 40 mg/L for 96 h at 15 °C. Controls consisted of aquarium water only. Hence, the exposure experiments consisted of 5 separate treatments. The fish were checked daily for any signs of discomfort or distress (erratic swimming behavior or immobilization) and immediately euthanized in 1 L of 50 mg/L MS222 buffered to pH 7.4 with NaHCO_3_ following recommendations of the animal care committee. The number of dead fish was recorded until the end of the 96-h period and the lethal concentration that kills 50% of fish (LC50) calculated using the Spearman-Karber method (Finney 1964). At the end of exposure period, the surviving fish were placed in 4 L of 20 mg/L MS222 at pH 7.4 for 5 min as described above for euthanasia.

The fish were immediately measured for mass (g) and head to tail length (cm) before placing on ice. The condition factor (CF) was obtained by the fish weight (g)/head to tail length (cm) ratio. The liver and gills were removed and weighted for the hepatic and gill somatic indexes (HSI and GiSI, respectively) and placed in 4–5 volumes of 140 mM NaCl containing 1 mM NaHCO_3_, 5 mM KH_2_PO_4_, and 1 µg/mL aprotinin (protease inhibitor) at 4 °C. They were homogenized using a Teflon pestle tissue grinder with 5 up-down passes. A portion of the homogenate was centrifuged at 12,000 × *g* for 20 min at 4 °C. The supernatant (S12 fraction) and the homogenates were stored at − 85 °C until analysis.

### Morphological biomarkers

The weight/length ratio was determined and expressed in g/cm. The GiSH and HSI were calculated by the organ weight/fish weight ratio. The relative levels of proteins were determined in the homogenate and S12 fraction using the Coomassie blue dye method (Bradford 1976). Briefly, the homogenates were diluted 1/20 in 5 mM NaOH and 5–10 µL was mixed to 160 µL with MilliQ water. Standard solutions of bovine serum albumin were used for calibration. Then, 40 µL of the Coomassie blue reagent (Bio-Rad, Ontario, Canada) was added to the samples, mixed, and the absorbance read at 595 nm in clear microplates (Neo-2 Synergy, Biotech instrument, CA, USA). The data were expressed as mg proteins/g liver or gills wet weight.

### Metallothionein-like proteins

The levels of heat stable metal binding proteins (mostly metallothioneins and glutathione) were determined by the Cd spectrophotometric assay for thioneins in the liver only (Suzuki et al. 1980). The principle of this assay resides on the increased absorbance at 254 nm when Cd binds to thiolates. The S12 fraction was first heat denatured at 100 °C for 5 min and centrifuged at 10,000 × *g* for 5 min at 4 °C. The supernatant was removed from the pellet and Cd^2+^ (prepared in MilliQ water) was added at 1 ppm for 15 min. The absorbance at 254 and 280 nm were determined in UV-transparent clear microplate. The blank consisted of the heat treated S_10_ without the addition of Cd. The data are expressed as A254 _S12+Cd_ -A254 _S12_ normalized to total proteins in the S12 fraction.

### Protein aggregation index

The extent of protein aggregation (amyloids) was determined using the fluorescence dye Thioflavine T in both liver and gills (Cabaleiro-Lago et al. 2010). Briefly, a 20 µL sample of S12 fraction was mixed with 200 µL of 10 µM of Thioflavine T (prepared in phosphate buffered saline) and fluorescence measured at 440 nm excitation and 480 nm emission in dark microplates (Neo-2, Synergy IV, Biotek Instruments, CA, USA). The data was expressed as relative fluorescence units/mg proteins in the S12 fraction.

### Biomarkers of damage

All the following biomarkers were performed in gills and liver. The levels of lipid peroxidation were determined using the thiobarbituric acid reactants (TBARS) methodology (Wills 1987). Calibration was achieved with solutions of tetramethoxypropane (stabilized form of malondialdehyde) and measured by fluorescence at 540-nm excitation and 590-nm emission in dark microplates (Neo-2, Synergy IV, Biotech Instrument, CA, USA). The data were expressed as ug TBARS/mg proteins. Finally, the levels of DNA damage (DNAd) were determined using the alkaline DNA precipitation assay using fluorescence detection (Gagné 2014). The principle of the assay consists of the precipitation of protein-bound genomic DNA from single- and double-stranded DNA following alkaline treatment in the presence of anionic detergent SDS. Following centrifugation at 8000 × *g* for 10 min, DNA strands remaining in solution were quantified by fluorescence using the Hoescht dye at 360-nm excitation and 460-nm emission in dark microplates (Neo-2, Synergy IV, Biotech Instrument, CA, USA). Standard solutions of salmon sperm DNA were used for calibration. The data were expressed as µg DNA/mg proteins in the homogenates.

### Data analysis

Each treatment was done in triplicates, and the exposure experiments repeated twice to confirm the toxicity responses. Biomarker data were obtained *N* = 8 trout for each treatment. The data normality and homogeneity of variance were confirmed by Shapiro–Wilk and Levene tests, respectively. The data were subjected to an analysis of variance followed by Fishers least square difference test to confirm significant (*p* > 0.05) changes in respect to controls. Toxicity data were expressed as threshold concentrations of Yb: (no effect concentration × effect concentration)^1/2^ in mg/L. The no-effect concentration was obtained by the highest concentration not statistically different from controls. Conversely, the effect concentration was the lowest concentration producing a significant difference from controls. Hierarchical tree analysis was also performed to seek out sublethal effects that were significantly associated to fish survival using the Pearson-moment correlation (1-*r*) for the distance between biomarker changes. Discriminant function analysis was also performed using fish survival as the predicted variables and to determine the most important biomarkers that best discriminates loss in survival (mortality). All statistical analyses were provided by the SYSTAT software package (version13).

## Results

### Toxicity and morphological changes

Yb is the 2^nd^ heaviest REE with a molecular weight of 173 g mol^−1^ and an ionic radius of 102 pm with relatively low electronegativity (1.1) (Emsley 1989). Yb has also the highest strong binding affinity to thiols with a log *K*_GSH_ = 7.68 (Garg et al. 2003). The LC50 of Yb was 2.7 ± 0.65 mg/L after 96 h at 15 °C. Survival gradually decreased with the exposure concentration: control (100% survival), 0.064 mg/L (100% survival), 0.32 mg/L (100% survival), 1.6 mg/L (60% survival), 8 mg/L (30% survival), and 40 mg/L (all dead, 0% survival). In the surviving fish, the fish weight/fork length (condition factor) and HSI were not significantly influenced at concentrations over the LC50 (8 mg/L) (Table 1). No changes in liver and gill protein contents were also observed. The GiSI was significantly reduced at the lowest concentration (0.06 mg/L).

**Table 1:** Morphological changes in rainbow trout juveniles exposed to ytterbium

Ytterbium (mg/L)	Condition factor	Hepatic somatic index (HIS)	Gill somatic index (GiSI)	Liver proteins	Gill proteins
Control	0.428 ± 0.008	0.012 ± 0.001	0.034 ± 0.002	0.187 ± 0.014	0.073 ± 0.005
0.06	0.432 ± 0.007	0.012 ± 0.001	0.027 ± 0.001*	0.189 ± 0.014	0.072 ± 0.003
0.32	0.426 ± 0.006	0.011 ± 0.001	0.027 ± 0.001*	0.181 ± 0.008	0.083 ± 0.003
1.6	0.425 ± 0.008	0.010 ± 0.001	0.030 ± 0.001	0.203 ± 0.024	0.070 ± 0.004
8	0.446 ± 0.014	0.011 ± 0.001	0.028 ± 0.001*	0.173 ± 0.009	0.063 ± 0.004

The asterisk symbol indicates significance at *p* < 0.05

### Hepatic thioneins levels

**Fig. 1:**
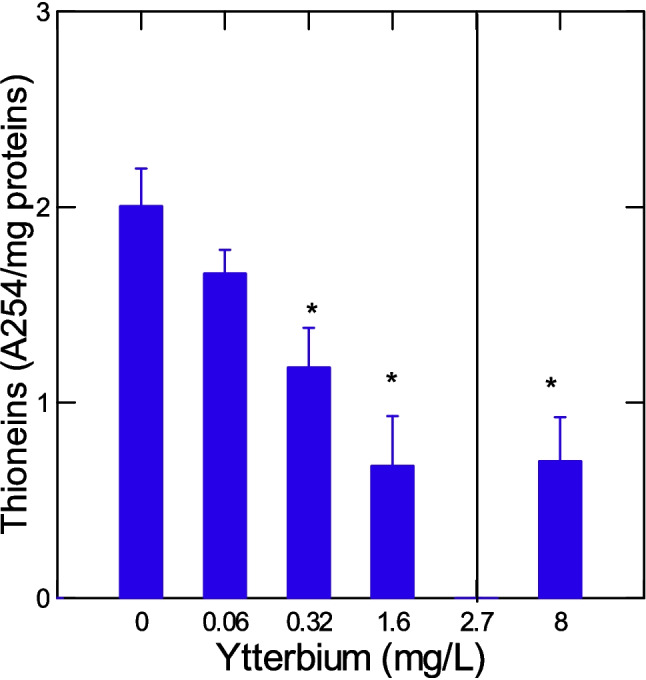
Changes in hepatic thioneins in trout juveniles exposed to ytterbium. The levels of metal-binding proteins were determined in the liver by the cadmium thioniein methodology. The data represent the mean with standard error of thioneins levels (A254 change/mg proteins). The line indicates the LC50 for Yb (2.7 ± 0.66 mg/L). The asterisk symbol indicates significance at *p* < 0.05 relative to controls

**Fig. 2:**
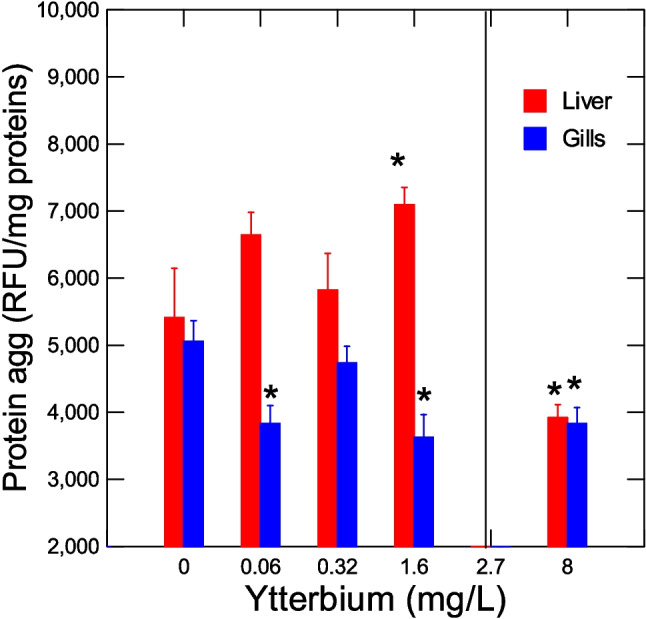
Amyloids levels in trout juveniles exposed to ytterbium. The levels of amyloids were determined in both the liver and gills. The data represent the mean with standard error. The line indicates the LC50 for Yb (2.7 ± 0.66 mg/L). The asterisk symbol indicates significance at *p* < 0.05 relative to controls

The levels of hepatic thioneins were significantly reduced at threshold concentration of 0.14 mg/L (Fig. 1). Thioneins were somewhat correlated with the number of surviving fish (*r* = 0.54), GiSI (*r* = 0.35), and HSI (*r* = 0.41). The levels of amyloids (protein aggregation) were determined in the liver and gills (Fig. 2). The levels were significantly increased in the liver at sublethal concentration of 1.6 mg/L just below the LC50 (2.7 mg/L). In gills, the levels were decreased at the lowest concentration (0.06 mg/L). Correlation analysis revealed that gills amyloids were correlated with GiSI (*r* = 0.46), survival (*r* = 0.51), thioneins (*r* = 0.37), and HSI (*r* = 0.37).

### Oxidative stress and DNA damage

**Fig. 3:**
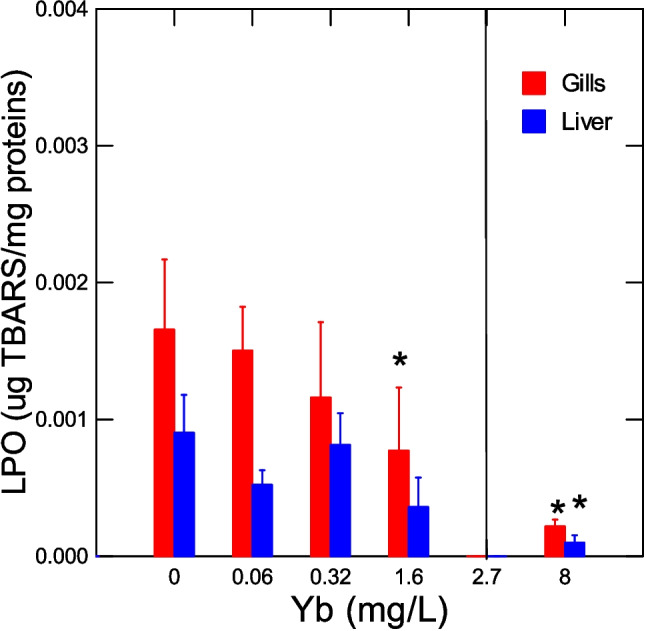
Lipid peroxidation in trout juveniles exposed to ytterbium. The LPO levels were determined in both the liver and gills. The data represent the mean with standard error. The line indicates the LC50 for Yb (2.7 ± 0.66 mg/L). The asterisk symbol indicates significance at *p* < 0.05 relative to controls

**Fig. 4:**
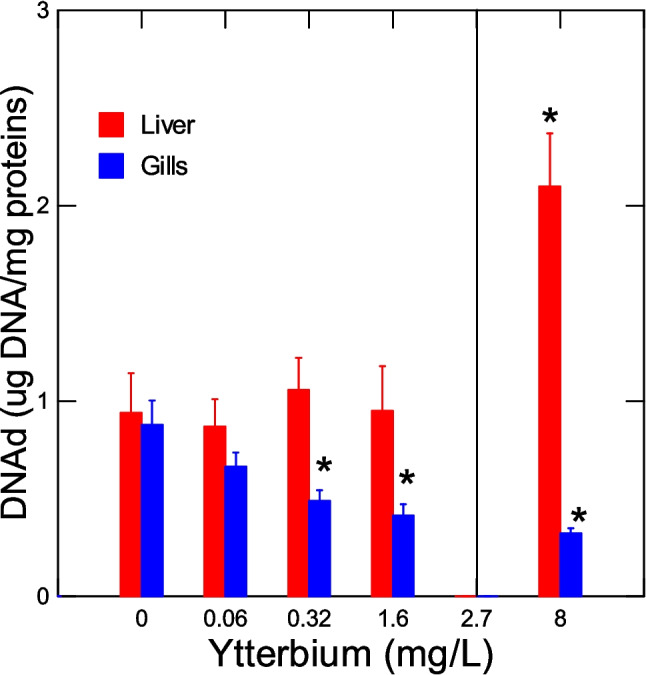
DNA damage in trout juveniles exposed to ytterbium. The levels of double -stranded breaks were determined in both the liver and gills. The data represent the mean with standard error of DNA damage (DNAd). The line indicates the LC50 for Yb (2.7 ± 0.66 mg/L). The asterisk symbol indicates significance at *p* < 0.05 relative to controls

The level of LPO were determined in the liver and gills (Fig. 3). Hepatic LPO dropped at a threshold concentration of 3.5 mg/L at the lethal concentration range and at a threshold concentration of 0.7 mg/L for LPO in gills. Correlation analysis revealed that liver LPO was significantly correlated with survival (*r* = 0.45), thioneins (*r* = 0.41), gill LPO (*r* = 0.4) and protein aggregation (amyloids) in gills (*r* = 0.49). Gill LPO was significantly correlated with survival (*r* = 0.48). The levels of DNA damage were determined in the liver and gills of trout juveniles (Fig. 4). In the liver, DNA strand breaks were significantly increased at the toxic threshold concentration of 4.6 mg/L above the LC50. In gills, the DNA strand breaks were significantly reduced at a threshold concentration of 0.14 mg/L below the LC50. Correlation analysis revealed that liver DNA strand breaks were significantly correlated with survival (*r* = − 0.44), liver proteins (*r* = − 0.61), and DNAd in gills (*r* = − 0.35). In gills, DNAd was significantly correlated with survival (*r* = 0.47), thioneins (*r* = 0.55), protein agg in gills (*r* = 0.52), liver LPO (*r* = 0.4), HSI (*r* = 0.41), and GiSI (*r* = 0.53).

### Multivariate analysis of biomarker responses

**Fig. 5:**
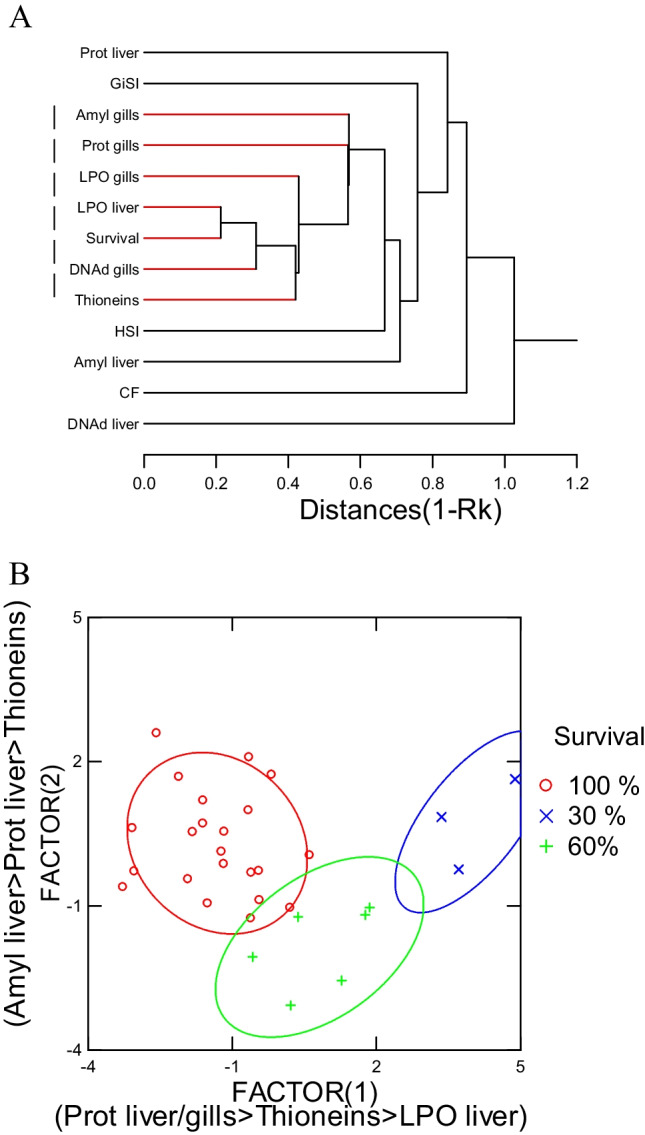
Hierarchical tree and discriminate function analyses of biomarkers with survival and morphological changes in trout juveniles exposed to ytterbium. Hierarchical tree (**A**) and discriminant function analysis based on fish survival (**B**) are shown. For tree analysis, the distance between the endpoints were determined by the rank correlation coefficient (1-Rk). The colored lines indicate significance at *p* < 0.05 from the correlation coefficient. The dotted line represents a cluster of biomarkers significantly related to survival (# of fish remaining following 96-h exposure). For discriminant analysis, the mean classification was 90%, and all the variance (100%) was explained by the 2 factors (axes). The controls correspond to 100% survival, while 30% survival indicates that the 30% of fish survived after 96-h exposure

In the attempt to gain a global understanding of the biomarker data in respect to decreased fish survival, a hierarchical tree and discriminant function analyses were performed (Fig. 5). Hierarchical tree analysis grouped the different biomarkers based on correlations and revealed the following biomarkers significantly related to the number of surviving fish: gill protein aggregation, liver and gills LPO, thioneins, gill DNA breaks and gill proteins (Fig. 5A). Discriminant function analysis was performed of fish survival (number of live fish remaining) instead of the exposure concentrations (Fig. 5B). The analysis revealed that a mean classification of 70% was achieved with 100% of the variance explained. The most important biomarkers separating the survival were similar to those found above: liver protein aggregation (amyloids), liver LPO, thioneins, and liver/gill proteins. This suggests that fish survival could be predicted by protein aggregation, LPO, and total proteins in gills and liver. This was confirmed by the significant multiple regression analysis of survival with these biomarkers (*R* = 0.71) with proteins in gills and liver, thioneins, and protein aggregation (amyloids).

## Discussion

### Interaction with DNA and proteins

Yb has the ability to bind biological ligands such as thiols, proteins, and DNA (Garg et al. 2003; Moodi et al. 2013; Aramesh-Boroujeni et al. 2020). Our data revealed that DNA strand breaks were increased in the liver at lethal concentrations and decreased in gills at sublethal concentrations. Phenanthroline complex of Yb showed binding activity towards DNA (Moodi et al. 2013). Indeed, the Yb-phenanthroline complex preferably binds to DNA grooves compared to phenanthroline. Moreover, Yb was shown to cleave DNA in the absence of reducing agents though to be responsible for its antimicrobial properties of Yb. This in keeping with the altered DNA breaks levels in the liver and gills of Yb exposed fish, especially for concentrations close to the LC50. While Yb reduced plasma levels of mitochondrial DNA copy number, it did not increase micronucleated circulating lymphocytes in a cohort study with occupational exposed workers (Hong et al. 2025). Yb-bipyridine complex also has affinity towards albumin (Aramesh-Boroujeni et al. 2020). This suggests that Yb could contribute to protein aggregation in addition to DNA breaks. A metallomic analysis of Yb and Lu on the model organism *Saccharomyces cerevisiae* revealed impacts on Ca^2+^-mediated transport processes and mitophagy, which is the process involving the degradation of mitochondria following damage (Pallares et al. 2023). Interestingly, these lanthanides target proteins of SH3 domains involved in the assembly of specific protein complexes for activation of various transduction pathways (Pawson and Schlessingert 1993). SH3 domains involved small protein (50 amino acids) found in a wide range of cytosolic and membrane proteins such as the noncatalytic domain of various tyrosine kinases. Yb also displays strong binding potential towards reduced glutathione (GSH) in the REEs family (Garg et al. 2003). It was previously suggested that REE with high affinity with GSH such as Lu and Tb reduced MT levels in fish hepatocytes (Hanana et al., 2021a, b). Conversely, REEs with low GSH binding were shown to induce MT and were generally more toxic to hepatocytes. This is consistent with the reduced thioneins in fish exposed to Yb in the present study. The toxicity of Yb was similar than Lu with a 96 h LC50 of 1.88 ± 0.9 mg/L (Hanana et al. 2021c). This corroborates another study with rainbow trout gill and *Danio rerio* hepatocyte cell lines; Yb and Lu were the most toxic REEs after 96 h based on cell viability (Fleurbaix et al. 2022).

### Induction of oxidative stress and damage

It was noteworthy that LPO was generally lower in both tissues in juvenile trout suggesting that Yb produced a reductive stress instead of oxidative stress. The enzyme catalase involved in the elimination of H_2_O_2_ was strongly inhibited by Yb exposure in the goldfish *Carassius auratus* (Hongyan et al. 2002). Inhibitions were observed at concentrations as low as 0.01 mg/L after 40 days of exposure time. The decrease in catalase activity was also accompanied by decreased superoxide activity suggesting decreased production of H_2_O_2_, indicating that oxygen radicals were not directly transferred to SOD/CAT pathways. However, fish survival was directly associated with LPO in both the liver and gills suggesting that decreased LPO is associated to reduced fish survival. Reduced LPO could be the result of reduced mitochondria activity by Yb. Indeed, about 1–3% of oxygen consumed by mitochondria during respiration is transformed to reactive oxygen radicals leading to H2O2 by the action of superoxide dismutase (Chance et al. 1979). In the liver, the subcellular distribution of radio-active Yb occurred mainly in the mitochondrial fraction (Ando et al. 1981) indicating that Yb targets mitochondria.

### Impacts on mitochondria function and metabolic activity

Yb^3+^ cause respiratory chain damage, membrane potential decrease, and reversed mitochondrial complex activity in rice mitochondria exposed in vitro (Gao et al. 2014). In another study, mitochondrial glutamate-pyruvate transaminase activity was inhibited at concentrations > 0.05 mg/L Yb after 40 days in goldfish suggesting decreased metabolic/mitochondrial activity (Hongyan et al. 2002). In the present study, LPO in the liver was significantly related to survival (*r* = 0.43) suggesting that lower LPO was associated with lower surviving fish, hence not a protective (antioxidant) effect of Yb. Exposure of rainbow trout to lutetium, its immediate neighbor in the periodic chart of elements, revealed decreased gene expression of mitochondrial cytochrome c oxidase following a 96-h exposure to 0.32 mg/L (Hanana et al. 2021a, b, c). Gene expression in SOD and CAT was also decreased at this concentration albeit marginally (0.05 < *p* < 0.1). This suggests that the more toxic heavy REEs sharing high GSH binding affinity involved depression in mitochondria activity and perhaps mitophagy. As with Yb, Lu decreased DNA strand breaks without the involvement of oxidative stress (LPO).

## Conclusions

In conclusion, Yb was toxic to rainbow trout juvenile with a 96 h LC50 of 2.7 ± 0.66 mg/L. Sublethal effects were observed at concentration as low as 0.06 mg/L, which was 45 times lower than the LC50. The following sublethal effects were significantly related to survival in trout juveniles: LPO, thioneins, gill DNA breaks, and protein aggregation (amyloids). The reported levels of Yb in municipal effluents are in the range of 5–30 ng/L (Turcotte et al. 2022), indicating that the toxicity of Yb within 96 h from municipal effluents is unlikely. On the one hand, this study sets the upper limits were Yb could initiate sublethal effects and for longer exposure times. On the other hand, since municipal wastewaters are considered continuous emitters of pollutants, including Yb, the bioavailability of this lanthanide should be monitored more closely in aquatic organisms near urban and mining pollution.

## Data Availability

Data will be made available upon request to the corresponding author.
